# Exploring the prevalence and characteristics of adverse drug events among older adults in South Korea using a national health insurance database

**DOI:** 10.3389/fphar.2022.1047387

**Published:** 2022-12-01

**Authors:** Eunkyeong Choi, Siin Kim, Hae Sun Suh

**Affiliations:** ^1^ College of Pharmacy, Pusan National University, Busan, South Korea; ^2^ Department of Pharmacy, Pusan National University Hospital, Busan, South Korea; ^3^ College of Pharmacy, Kyung Hee University, Seoul, South Korea; ^4^ Department of Regulatory Science, Graduate School, Kyung Hee University, Seoul, South Korea; ^5^ Institute of Regulatory Innovation Through Science, Kyung Hee University, Seoul, South Korea

**Keywords:** adverse drug event, prevalence, drug safety, elderly, female, diagnosis codes

## Abstract

**Background:** Adverse drug events (ADEs) in the elderly frequently occur because of their multiple chronic diseases and complexity of drug therapy. To better understand adverse drug events, the prevalence and characteristics of adverse drug events in elderly South Korean patients were assessed.

**Methods:** The National Health Insurance databases for 2015 and 2016 were used for the analysis. We included patients aged ≥65 years that had at least one claim with the diagnosis codes ‘drug-induced,’ ‘poisoning by drug,’ and ‘vaccine-associated’ each year for the base-case analysis. To minimize the underestimation of adverse drug event prevalence, we also used an extended definition analysis by adding the ‘adverse drug event very likely’ codes. We estimated the prevalence of adverse drug events by sex, age group, and type of insurance and examined the frequent types of adverse drug events in 2015 and 2016.

**Results:** In the base-case analysis, adverse drug event prevalence in individuals aged 65 years and older was 2.75% in 2015 and 2.77% in 2016. With advanced age, the prevalence of adverse drug event tended to increase, peaking in the age group of 75–79 years. In addition, the adverse drug event prevalence was higher in females and Medical Aid enrollees. The most frequently occurring adverse drug event was ‘allergy, unspecified,’ followed by ‘other drug-induced secondary parkinsonism,’ and ‘generalized skin eruption due to drugs and medicaments.’ When we examined the extended definition analysis, the prevalence of adverse drug events was 4.47% in 2015 and 4.52% in 2016, which significantly increased from those estimated in the base-case analysis.

**Conclusion:** Among the older adults, the prevalence of adverse drug event was higher in advanced age, females, and Medical Aid enrollees. In particular, allergy and drug-induced secondary parkinsonism frequently occurred. This study provides evidence that health policies addressing the prevention and management of adverse drug events should be a priority for the most vulnerable elderly patients.

## Introduction

Adverse drug events (ADE) are untoward complications that may occur during drug therapy (Nebeker et al., 2004). Bates et al. defined an ADE as any injury resulting from drug-related medical interventions, including medication errors (Bates et al., 1997). ADE is a broad spectrum of definitions compared with an adverse drug reaction (ADR), which is harmful and unintended consequences, occurring at appropriate use of drugs (Nebeker et al., 2004). Because nearly half of ADEs come from medication errors and can be prevented, only considering the effect of medications normally used underestimates the problem (Bates et al., 1995; Lghoul-Oulad Saïd et al., 2020).

ADEs are an essential public health issue that contribute to morbidity and a considerable economic burden on healthcare resources (Bates et al., 1995; Bates et al., 1997; Classen et al., 1997). According to a review of forty-seven European studies, hospital admissions due to ADRs, a subset of ADEs, were 3.6%, and the occurrence of ADRs during the hospital stay was 10.1% (Bouvy et al., 2015). The costs associated with ADEs in two tertiary care hospitals were estimated at $5.6 million annually, even in the late 1990s (Bates et al., 1997).

In particular, elderly patients are at high risk of ADEs because they have altered drug metabolism, have more chronic diseases, and take several medications (Field et al., 2004; Pedrós et al., 2014). For example, in South Korea, 86.4% of those aged 65 years or above had polypharmacy, defined as the concurrent use of six or more medications per person (Kim H. et al., 2014). Furthermore, a large meta-analysis reported that hospital admission related to ADR in the elderly was four times higher than in younger adults (Beijer and de Blaey, 2002). Therefore, efforts to improve patient safety in the elderly by reducing ADEs are a public health priority (Bates et al., 2009).

Despite the widespread recognition that ADEs are common in elderly patients and extensive epidemiological studies being conducted in Western countries (Field et al., 2004; Passarelli et al., 2005; Alhawassi et al., 2014; Friedman et al., 2015), the prevalence of ADEs and their characteristics have not been well described in the Asian population, including those in South Korea (Leendertse et al., 2010). Moreover, although a few studies have estimated the prevalence of ADEs in South Korea using medical chart reviews and spontaneous reporting (Koo, 2009; Shin et al., 2009; Yu et al., 2015), these studies lack generalizability because the study populations were limited. Several studies have suggested that claims data provide a complementary and alternative method for detecting ADEs with other monitoring systems, such as chart reviews, voluntary reporting, and computerized surveillance (Hougland et al., 2006; Miguel et al., 2013; Kuklik et al., 2017; Digmann et al., 2019). South Korea has a single National Health Insurance program; all populations are covered under this program, approximately 50 million people. The Health Insurance Review and Assessment (HIRA) database contains not only individual insurance information but various health information, including diseases, symptoms, and prescribed medication (Kim et al., 2017). It provides healthcare coverage to all outpatient and inpatient services. Therefore, we conducted this population-based study using a National Health Insurance database to assess the prevalence of ADEs in elderly patients and identify the types of ADEs that occurred in South Korea. We compared the annual prevalence of ADEs and examined patterns of prevalence by sex, age group (65–69, 70–74, 75–79, and ≥80 years), and type of insurance.

## Materials and methods

### Data source

We conducted a descriptive, retrospective study using Health Insurance Review and Assessment Service-National Patient Sample (HIRA-NPS) claims data from 2015 to 2016. The HIRA-NPS claims data are available from the Health Insurance Review and Assessment Service through a formal request for research purposes (Kim et al., 2017). The HIRA-NPS data are designed to approximate a 3% stratified sample (approximately 1,400,000 persons) of the entire population enrolled in the National Health Insurance (NHI) or Medical Aid (MA) program each year (Kim H et al., 2014). The Patient Sample data was generated systematically by probabilistic sample extraction method using stratified sampling with a total of 32 strata based on sex (2 strata) and age (16 strata) (Kim H et al., 2014). South Korea has a government-run mandatory national health security program consisting of NHI and MA program enrollees. The NHI program is a wage-based, contributory insurance program covering approximately 96% of the population, while the MA program is a government-subsidized public assistance program for low-income and medically indigent individuals (Song, 2009). The patient sample database confirmed the representativeness of the entire South Korean population through a validity test (Kim et al., 2013).

The HIRA-NPS data are cross-sectional, and different patients were selected for the sample data each year for their privacy; therefore, it is not possible to follow an individual over the years (Kim et al., 2017). The data contain each patient’s unique encrypted identification number, age, sex, type of insurance, diagnosis, and prescription drugs, which provide valuable resources for healthcare service research (Kim L et al., 2014b). Diagnoses were encoded in accordance with the *International Classification of Diseases, 10th Revision* (ICD-10).

### Study participants and definition of adverse drug events

To be included in this study, participants with ADEs needed to be aged ≥65 years and have at least one NHI or MA claim record of outpatient, inpatient, or emergency department services with an ADE diagnosis code from the HIRA-NPS in 2015–2016. According to the prevalence-based approach, patients had both new and pre-existing cases of ADEs each year (Kim Y et al., 2013).

We selected diagnosis codes for ADEs from a previous systematic review to identify ADEs in the claims data (Hohl et al., 2014). The ADE diagnosis codes include the phrase or meaning ‘drug-induced,’ ‘poisoning by drug,’ and ‘vaccine-associated.’ These codes directly describe the drug’s relevance to a symptom or disease.

Furthermore, to minimize the underestimation of the prevalence of ADEs, we added ‘ADE very likely’ codes to comprehensively capture ADEs from the claim records (Hohl et al., 2014). Diagnosis codes associated with ‘ADE very likely’ do not refer to a drug in the diagnosis code description. However, they are probably associated with drug use, according to a causality assessment by clinical experts in a previous study (Hohl et al., 2014). For the analyses, 586 codes, together with sub-codes, were used to identify ADEs, including ‘drug-induced,’ ‘poisoning by drug,’ ‘vaccine-associated,’ and ‘ADE very likely.’ The diagnosis codes and descriptions of the ADEs are presented in Table 1 and Supplementary Table S1, respectively.

**TABLE 1:** Definitions of diagnosis codes and examples of adverse drug events.

Classification	Definition	Examples of diagnosis codes^1^ ^)^	
		ICD-10 code	Description
Drug-induced	The ICD-10 code description includes ‘induced by drug’	G25.1	Drug-induced tremor
	The ICD-10 code description includes ‘induced by drug or other causes’	I42.7	Cardiomyopathy due to drugs and other external agents
Poisoning by drug	The ICD-10 code description includes ‘poisoning by drug’	T36	Poisoning by systemic antibiotics
	The ICD-10 code description includes ‘poisoning by or harmful use of a drug or other causes’	F55	Abuse of non-dependence-producing substances
Vaccine-associated	Vaccine-associated adverse event	A80.0	Acute paralytic poliomyelitis, vaccine-associated
ADE very likely	Adverse drug event deemed to be very likely although the ICD-10 code description does not refer to a drug	A04.7	Enterocolitis due to *Clostridium difficile*

ADE, adverse drug event; ICD-10, *International Classification of Diseases, 10th Revision*.

^1^ A full list of diagnosis codes is listed in Supplementary Material.

### Statistical analysis

We estimated the annual prevalence of ADE as the number of patients with ADEs divided by the number of the entire HIRA-NPS population each year. The results were expressed as frequency and percentage (%). To evaluate whether the ADE prevalence had changed annually, the differences in the prevalence of ADEs between 2015 and 2016 were analyzed using the Cochran–Armitage trend test.

To better understand patient characteristics associated with the occurrence of ADEs, we also calculated the age-and sex-specific prevalence in each year and compared the prevalence stratified by sex (male, female), age group (65–69, 70–74, 75–79, and ≥80), and type of insurance (NHI, MA) between 2015 and 2016. In order to compare the sex differences, we calculated the female-to-male ratio of prevalence by age group. Chi-square tests were used to compare differences in prevalence between the sexes.

Additionally, we identified the characteristics of ADE each year and compared the differences between the sexes. To determine the frequent types of ADEs, the frequency of each diagnosis code to define ADEs was calculated annually in 2015 and 2016. If a diagnosis code was given repeatedly to a patient, the code was considered to be claimed only once.

Sensitivity analysis was performed to compare the two approaches in defining the patients with ADE. The patients in the base-case group have the diagnosis codes of ‘drug-induced,’ ‘poisoning by drug,’ and ‘vaccine-associated.’ The patients in the extended definition group included base-case patients and those who have the diagnosis codes of ‘ADE very likely.’ The chi-square test was used to compare the results of the base-case and extended definition analyses.

Statistical analysis was performed using the SAS software (version 9.4; SAS Institute Inc., Cary, NC, United States). Statistical significance was set at *p* ≤ 0.05.

## Results

Based on the base-case analysis, 5,257 and 5,515 patients aged 65 years and older were identified as having ADEs in 2015 and 2016, respectively (Table 2). The number of adverse events identified in the claims records was 5,500 and 5,748 in 2015 and 2016, respectively.

**TABLE 2:** Prevalence of adverse drug events by sex, age group, and type of insurance.

	2015			2016			*p*-value^1^ ^)^
	Male	Female	Overall	Male	Female	Overall	
No. Claims	6,914	12,311	19,225	7,359	11,809	19,168	
No. Events	2,112	3,388	5,500	2,253	3,495	5,748	
No. Patients	2,012	3,245	5,257	2,171	3,344	5,515	
Prevalence (%)							
Overall	2.53	2.91	2.75	2.61	2.89	2.77	0.6460
Age group							
65–69	2.18	2.88	2.54	2.43	2.94	2.69	0.1015
70–74	2.69	2.93	2.82	2.64	2.92	2.80	0.8088
75–79	2.86	3.35	3.15	2.79	3.26	3.07	0.4899
≥80	2.63	2.51	2.55	2.74	2.49	2.57	0.8556
Type of insurance							
NHI program	2.49	2.84	2.69	2.57	2.84	2.72	0.5054
MA program	3.28	3.68	3.56	3.29	3.45	3.40	0.4548

NHI, national health insurance; MA, medical aid.

^1^ *p*-value from Cochrane-Armitage test for trend of overall prevalence.

**FIGURE 1:**
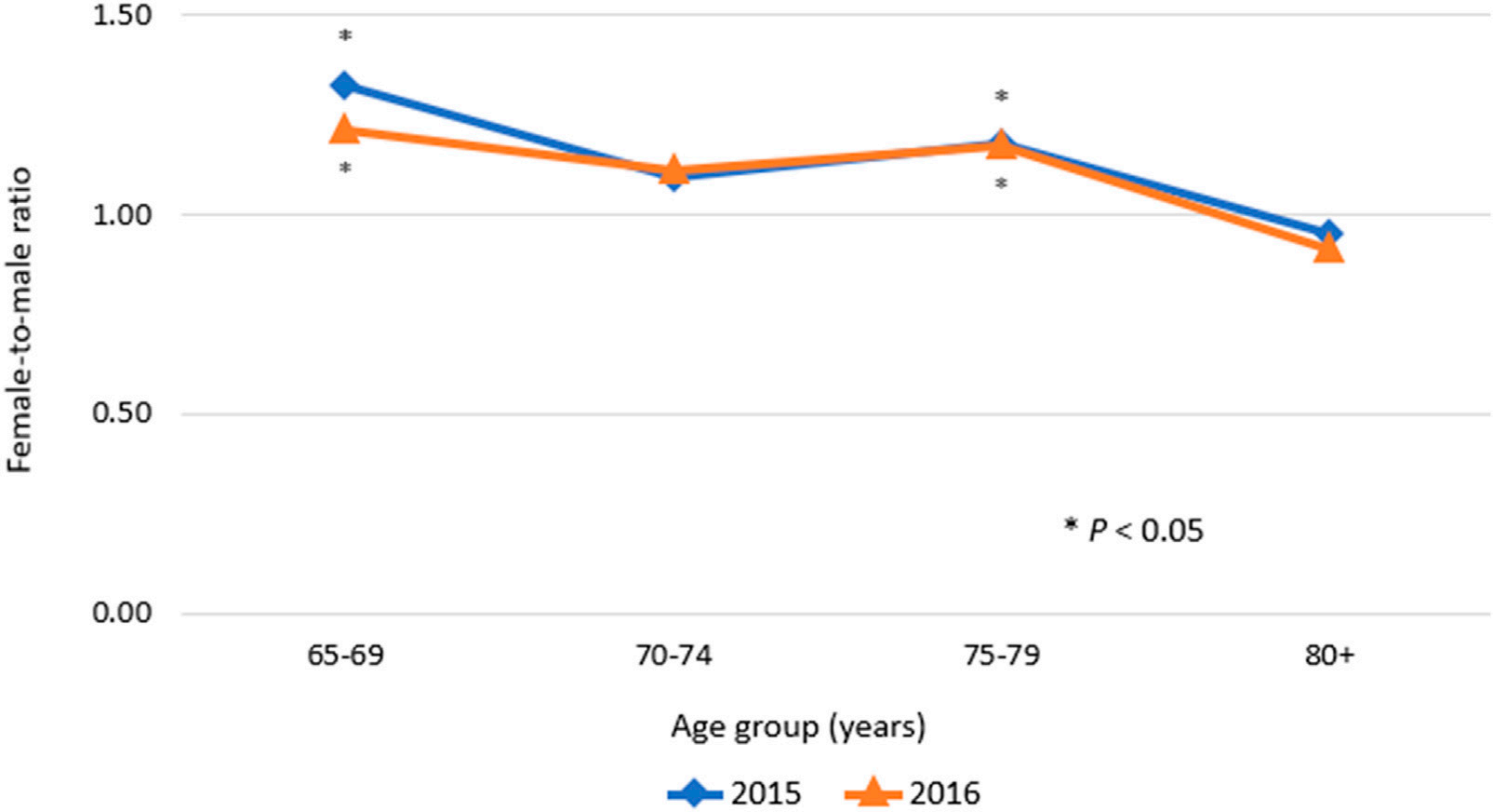
Female-to-male ratio of prevalence of adverse drug event.

The prevalence of ADEs was 2.75% and 2.77% in 2015 and 2016, respectively. There was no significant difference in overall prevalence between calendar years. In all age groups and types of insurance, the trends in the annual prevalence were quite similar. In both 2015 and 2016, a higher prevalence was observed with increasing age, with the peak prevalence observed in the age group of 75–79 years and a higher prevalence in females. The female-to-male ratio of prevalence of ADEs was significantly higher than 1.0 in the age group of 65–69 years and the age group of 75–79 years (*p* < 0.05; Figure 1). In contrast, in the over-80 age group, a higher prevalence of ADEs was observed in males compared with females. In addition, in both men and women, the prevalence of ADEs was higher in MA program enrollees compared with NHI program enrollees.

**FIGURE 2:**
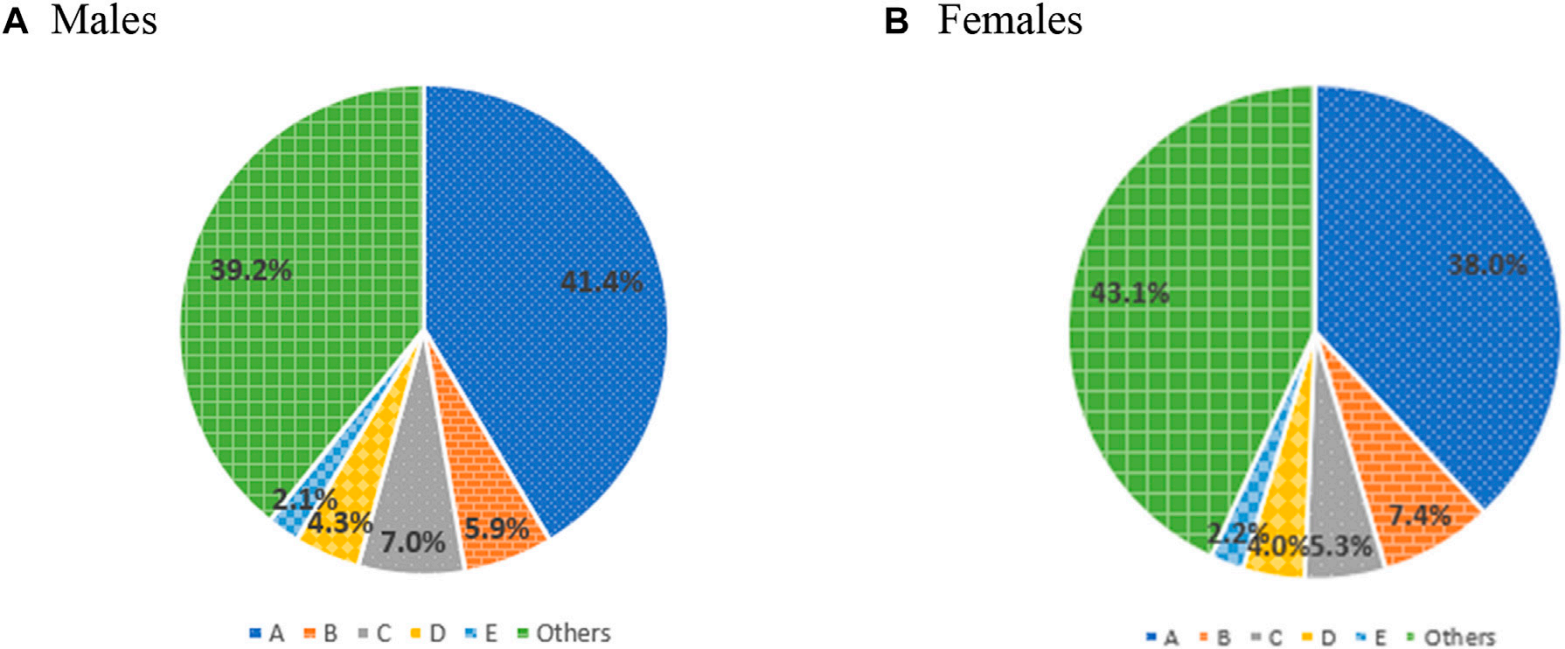
Prevalence of frequent type of adverse drug events by sex. A, allergy, unspecified; B, other drug-induced secondary parkinsonism; C, generalized skin eruption due to drugs and medicaments; D, allergic contact dermatitis due to drugs in contact with skin; E, unspecified adverse event of drug or medicament.

During the study period, the most common ADEs were ‘allergy, unspecified,’ followed by ‘other drug-induced secondary parkinsonism,’ and ‘generalized skin eruption due to drugs and medicaments’ (Table 3). The patterns in the characteristics and frequencies of ADEs were comparable to 2015 and 2016. Notably, ‘other drug-induced secondary parkinsonism,’ the second most common ADE, illustrated a higher distribution in females than in males (Figure 2).

**TABLE 3:** Most frequent type of adverse drug events in 2015–2016.

ICD-10 codes	Code description	No. Events (%)			
		2015		2016	
T78.4	Allergy, unspecified	2,156	(39.20)	2,261	(39.34)
G21.1	Other drug-induced secondary parkinsonism	333	(6.05)	391	(6.80)
L27.0	Generalized skin eruption due to drugs and medicaments	312	(5.67)	343	(5.97)
L23.3	Allergic contact dermatitis due to drugs in contact with skin	247	(4.49)	239	(4.16)
T88.7	Unspecified adverse event of drug or medicament	178	(3.24)	183	(3.18)
E27.3	Drug-induced adrenocortical insufficiency	164	(2.98)	163	(2.84)
L27.1	Localized skin eruption due to drugs and medicaments	148	(2.69)	166	(2.89)
E24.2	Drug-induced Cushing’s syndrome	146	(2.65)	151	(2.63)
Y45.3	Drugs, medicaments and biological substances causing adverse effects in therapeutic use - Analgesics, antipyretics and anti-inflammatory drugs	133	(2.42)	132	(2.30)
T78.2	Anaphylactic shock, unspecified	121	(2.20)	140	(2.44)
T78.3	Angioneurotic oedema	113	(2.05)	125	(2.17)
L27.9	Dermatitis due to unspecified substance taken internally	108	(1.96)	114	(1.98)
G25.1	Drug-induced tremor	106	(1.93)	104	(1.81)
M81.4	Drug-induced osteoporosis without pathological fracture	92	(1.67)	102	(1.77)
L27.8	Dermatitis due to other substances taken internally	79	(1.44)	87	(1.51)
L24.4	Irritant contact dermatitis due to drugs in contact with skin	58	(1.05)	70	(1.22)
Others		1,006	(18.29)	977	(17.00)
Total		5,500	(100)	5,748	(100)

ICD-10, *International Classification of Diseases, 10th Revision*.

Using the extended definition of ADEs to minimize underestimation, the prevalence of ADEs increased significantly (*p* < 0.0001; Table 4). According to the extended definition analysis, the prevalence of ADE was 4.47% and 4.52% in 2015 and 2016, respectively, which increased by approximately 60% compared to the estimates of base-case analysis.

**TABLE 4:** Prevalence of adverse drug events by definition study population.

	Base-case		Extended definition		*p*-value^1^ ^)^
	2015	2016	2015	2016	
No. Claims	19,225	19,168	27,878	28,401	
No. Events	5,500	5,748	9,156	9,607	
No. Patients	5,257	5,515	8,556	8,991	
Prevalence (%)	2.75	2.77	4.47	4.52	<0.0001

^1^ *p*-value from chi-square test between base-case and extended-definition estimates.

## Discussion

In this study, we estimated the prevalence of ADEs in people aged 65 years or older in South Korea using the nationally representative claims data. We also examined whether the prevalence of ADEs changed by comparing the prevalence each year and identified the types of ADEs that occurred during the study period.

Based on the HIRA-NPS database, the base-case analysis in our study found that the estimated prevalence of ADEs for those aged 65 years or above in 2015 and 2016 were relatively lower than those reported in other countries. A study using data from a national survey in the United States reported that visits to emergency departments and outpatient clinics related to ADEs were 48.8 per 1,000 persons between 2001 and 2005 (Bourgeois et al., 2010). A systematic review that included fourteen observational studies reported that the prevalence of ADR, a subset of ADE, was 11.0%, ranging from 5.8% to 46.3% (Alhawassi et al., 2014). However, our estimates are significantly higher compared with a previous study that reported ADE prevalence among patients aged 65 years and older who visit an emergency department in a tertiary-care hospital in South Korea was 0.45% (Lee, 2015).

Based on a previous systematic review of sixty-eight studies (Beijer and de Blaey, 2002), our results supported that the prevalence of ADEs increases with age. This is in line with a recent systematic review of thirty-three studies that reported patients aged ≥65 years showed the highest prevalence of ADEs (Insani et al., 2021). Because elderly patients usually have many underlying diseases leading to polypharmacy, they are at risk of ADEs (Hajjar et al., 2007; Atella et al., 2019; Khezrian et al., 2020). According to a previous study, the potential preventability of hospital admission related to medication in elderly patients was approximately twice that in younger patients (Lghoul-Oulad Saïd et al., 2020). Therefore, elderly patients are imperative target populations for effective intervention strategies to prevent ADEs.

Concerning sex differences, we observed that females had a significantly higher prevalence of ADEs than males. This finding is consistent with the results of several other studies (Martin et al., 1998; Zopf et al., 2008; Bourgeois et al., 2010; Kane-Gill et al., 2010; Hofer-Dueckelmann et al., 2011; Wu et al., 2012). For example, an observational study including all patients admitted to an internal hospital in Austria over 6 months reported that more females than males experienced ADEs, particularly elderly (10% vs 6%, *p* < 0.005) (Hofer-Dueckelmann et al., 2011). The potential reasons for the differences in ADE prevalence by sex can be explained by differences in pharmacokinetics, pharmacodynamics, and drug utilization patterns (Tran et al., 1998; Zucker and Prendergast, 2020).

Patients enrolled in the MA program had a higher prevalence of ADEs than those in the NHI program. This might be related to excessive healthcare resource use and polypharmacy among MA program enrollees (Kim H. et al., 2014; Suh et al., 2014). Previous studies comparing individuals with NHI coverage and those with MA coverage for healthcare utilization revealed that MA program enrollees showed more frequent outpatient visits and hospital admissions (Lee et al., 2020). In addition, Kim et al. reported significant associations between polypharmacy and the lower-income MA population (Kim L et al., 2014). A possible reason for the excessive use of medical services and polypharmacy in the MA program enrollment is that they are not required to provide co-payments for almost all healthcare utilization (Suh et al., 2014; Lee et al., 2020). Furthermore, previous studies have reported that polypharmacy is a significant risk factor for ADEs because of the increased possibility of drug-drug interactions and inappropriate drug use (Onder et al., 2002; Field et al., 2004; Steinman et al., 2006; Rashed et al., 2012). Therefore, quality improvement, such as drug utilization review programs, is recommended to prevent meaningful drug-drug interactions and duplicate prescriptions (Aparasu et al., 2005).

In the present study, allergy and skin manifestations were the most frequent ADEs identified in the claims data. This finding is consistent with a previous result based on a spontaneous report conducted in South Korea (Shin et al., 2009). However, the characteristics of ADEs in our study differed from those in other countries. For example, in a retrospective study examining ADR-related hospital admissions at a single hospital in Thailand, ‘drug-induced neutropenia’ was the most common (Siltharm et al., 2017). Another study conducted in England, Germany, and the United States revealed that ‘Enterocolitis due to *Clostridium difficile*’ was the most frequent type of ADEs (Stausberg, 2014).

Notably, the second most common ADE was ‘other drug-induced secondary parkinsonism,’ which more frequently occurred in females. An observational study in South Korea reported that females and the elderly showed a high prevalence and incidence of drug-induced secondary parkinsonism (Han et al., 2019). Antipsychotics and gastrointestinal motility drugs are frequently associated with drug-induced secondary parkinsonism (Shin and Chung, 2012; López-Sendón et al., 2013; Kim et al., 2019; Kim and Suh, 2019). It is essential to bear in mind that physicians and other healthcare providers frequently overlook the presence of drug-induced parkinsonism because it is challenging to differentiate drug-induced parkinsonism from idiopathic Parkinson’s disease (Hansen et al., 1992). Recovery after the withdrawal of causal drugs may take several years, and clinical deficits might be progressive and persistent in some cases. Therefore, based on our results, effective intervention to prevent drug-induced secondary parkinsonism would be a critical component of ADE management for the elderly.

Because ADEs are expressed as various signs, symptoms, or diseases, it is difficult to identify an ADE based on the diagnosis codes from the claims record. Therefore, to improve the detection of ADE cases, two approaches by different ADE definitions were used in this study: the base-case and extended definition groups. From the base-case analysis of patients, the number of patients identified according to the extended definition of ADEs increased significantly. In a previous study, the reported prevalence of ADEs varied depending on the operational definition of events, as well as specific aspects such as the study setting, study population, and data collection methods (Leendertse et al., 2010). Stausberg and Hasford studied the prevalence of ADEs using more broad definitions of diagnosis codes, including ‘ADE likely’ and ‘ADE possible,’ which were less associated with drug use than ‘ADE very likely.’ According to their definitions of ADEs, the prevalence of drug-related hospital admission and hospitalization considerably differed, ranging from less than 1%–37.6% (Stausberg and Hasford, 2011). However, data estimates are possibly uncertain because validity and reliability could not be assessed owing to limited information from the claims data; thus, careful interpretation is needed to understand these results.

To the best of our knowledge, this study provides the first comprehensive estimate of the prevalence of ADEs in the elderly in the Asian population using claims data. Our results are representative because the HIRA-NPS claims data provide reliability, and valid information for the entire population of South Korea. Furthermore, various ADEs are identified through the broader focus of adverse events, including the consequences of inappropriate drug use, even though our results are conservative because study participants are limited due to our operational definitions of ADEs.

This study had several limitations, including the potential underestimation of ADEs. First, not all ADEs could be identified due to the limitations of the diagnosis codes. Not all ADEs can be searched using the codes, including the phrases ‘drug-induced,’ ‘poisoning by drug,’ ‘vaccine-associated,’ or even ‘ADE very likely,’ because ADE codes could not cover all potential illnesses or symptoms caused by drugs. Moreover, patient-reported adverse events or abnormalities in laboratory results related to drugs were not recorded because of the limited clinical information available in the claims data. Second, physician under-reporting could account for the low estimate of ADE prevalence. Although physicians are obligated to monitor patients’ ADEs during their practice, a significant proportion do not report ADEs (Dormann et al., 2003). Several reasons for not reporting ADEs include a lack of time due to stressful environments, uncertainty about the drug causing the ADE, difficulty in accessing reporting systems, and lack of awareness of the need to record that ADEs have occurred (Hazell and Shakir, 2006). Third, we included only study participants with at least one claim-encoded ADE diagnosis code. However, most ADEs are mild; thus, a substantial number of patients may not seek medical care for minor signs or symptoms caused by drugs. Fourth, the possible drugs associated with ADEs could not be determined using claims data because of the limited clinical information available and the retrospective study design. Fifth, the 2-year study period may be insufficient to understand any trends in ADE prevalence. Sixth, we did not analyze ADE prevalence in different clinical settings. Further studies are needed to understand the prevalence of ADE in outpatient, inpatient, and emergency departments. Lastly, it was not recent data that we used in this study. Therefore, our study results may not reflect current estimates. However, after 2016, several diagnosis codes classified as sensitive information were not provided in the HIRA-NPS database. Therefore, we used the 2015 and 2016 database, the most recent database that we could fully identify all diagnosis codes.

## Conclusion

This study using the representative claims data provided comprehensive estimates of ADE prevalence and characteristics among the elderly in South Korea. Due to the extended life expectancy, the prevalence of ADEs is expected to grow continuously. The results of our study suggest that more efforts will be needed to prevent ADE in the elderly. National healthcare policy, such as regulatory intervention for polypharmacy and educational program, is required to reduce ADE for vulnerable people, especially in MA program enrollees. To effectively prevent and manage ADEs, further studies are needed to explore potential drugs that cause ADEs.

## Data Availability

The datasets presented in this article are not readily available because this study used the Health Insurance Review and Assessment Service-National Patient Sample (HIRA-NPS) 2015 and 2016 database in South Korea. Data were obtained from the HIRA with permission.
