# Supplementary material for: Exploring the prevalence and characteristics of adverse drug events among older adults in South Korea using a national health insurance database
Source: Front Pharmacol. 2022 Dec 1;13:1047387. doi: 10.3389/fphar.2022.1047387 (PMC9751698; doi:10.3389/fphar.2022.1047387)
Supplement: Supplementary file 1 [file Table1.DOCX]

Table S1. List of the *International Classification of Diseases, Tenth Revision* (ICD-10) codes associated with adverse drug events

| **ICD-10 code** | **Description** |
| --- | --- |
| ***Drug-induced*** | |
| D52.1 | Drug-induced folate deficiency anaemia |
| D59.0 | Drug-induced autoimmune haemolytic anaemia |
| D59.2 | Drug-induced nonautoimmune haemolytic anaemia |
| D61.1 | Drug-induced aplastic anaemia |
| D64.2 | Secondary sideroblastic anaemia due to drugs and toxins |
| D68.3 | Hemorrhagic disorder due to circulating anticoagulants |
| E03.2 | Hypothyroidism due to medicaments and other exogenous substances |
| E06.4 | Drug-induced thyroiditis |
| E16.0 | Drug-induced hypoglycaemia without coma |
| E23.1 | Drug-induced hypopituitarism |
| E24.2 | Drug-induced Cushing’s syndrome |
| E27.3 | Drug-induced adrenocortical insufficiency |
| E66.1 | Drug-induced obesity |
| F11* | Mental and behavioural disorders due to use of opioids |
| F13* | Mental and behavioural disorders due to use of sedatives or hypnotics |
| F15* | Mental and behavioural disorders due to use of other stimulants, including caffeine |
| F19* | Mental and behavioural disorders due to multiple drug use and use of other psychoactive substances |
| G21.0 | Malignant neuroleptic syndrome |
| G21.1 | Other drug-induced secondary parkinsonism |
| G21.2 | Secondary parkinsonism due to other external agents |
| G24.0 | Drug-induced dystonia |
| G25.1 | Drug-induced tremor |
| G25.4 | Drug-induced chorea |
| G25.6 | Drug-induced tics and other tics of organic origin |
| G44.4 | Drug-induced headache, not elsewhere classified |
| G62.0 | Drug-induced polyneuropathy |
| G72.0 | Drug-induced myopathy |
| H26.3 | Drug-induced cataract |
| H40.6 | Glaucoma secondary to drugs |
| I42.7 | Cardiomyopathy due to drugs and other external agents |
| I95.2 | Hypotension due to drugs |
| J70.2 | Acute drug-induced interstitial lung disorders |
| J70.3 | Chronic drug-induced interstitial lung disorders |
| J70.4 | Drug-induced interstitial lung disorders, unspecified |
| K85.3 | Drug-induced pancreatitis |
| L10.5 | Drug-induced pemphigus |
| L23.3 | Allergic contact dermatitis due to drugs in contact with skin |
| L24.4 | Irritant contact dermatitis due to drugs in contact with skin |
| L25.1 | Unspecified contact dermatitis due to drugs in contact with skin |
| L27.0 | Generalized skin eruption due to drugs and medicaments |
| L27.1 | Localized skin eruption due to drugs and medicaments |
| L27.8 | Dermatitis due to other substances taken internally |
| L27.9 | Dermatitis due to unspecified substance taken internally |
| L43.2 | Lichenoid drug reaction |
| L56.0 | Drug phototoxic response |
| L56.1 | Drug photoallergic response |
| L64.0 | Drug-induced androgenic alopecia |
| M10.2 | Drug-induced gout |
| M32.0 | Drug-induced systemic lupus erythematosus |
| M34.2 | Systemic sclerosis induced by drugs and chemicals |
| M80.4 | Drug-induced osteoporosis with pathological fracture |
| M81.4 | Drug-induced osteoporosis without pathological fracture |
| M83.5 | Other drug-induced osteomalacia in adults |
| M87.1 | Osteonecrosis due to drugs |
| N14.0 | Analgesic nephropathy |
| N14.1 | Nephropathy induced by other drugs, medicaments and biological substances |
| N14.2 | Nephropathy induced by unspecified drug, medicament or biological substance |
| N14.4 | Toxic nephropathy, not elsewhere classified |
| O35.5 | Maternal care for (suspected) damage to fetus by drugs |
| O74.4 | Toxic reaction to local anaesthesia during labour and delivery |
| P04.0 | Fetus and newborn affected by maternal anaesthesia and analgesia in pregnancy, labour and delivery |
| P04.1 | Fetus and newborn affected by other maternal medication |
| P04.4 | Fetus and newborn affected by maternal use of drugs of addiction |
| P58.4 | Neonatal jaundice due to drugs or toxins transmitted from mother or given to newborn |
| P93 | Reactions and intoxications due to drugs administered to fetus and newborn |
| P96.1 | Neonatal withdrawal symptoms from maternal use of drugs of addiction |
| P96.2 | Withdrawal symptoms from therapeutic use of drugs in newborn |
| Q86.1 | Fetal hydantoin syndrome |
| Q86.2 | Dysmorphism due to warfarin |
| R50.2 | Drug-induced fever |
| T78.2 | Anaphylactic shock, unspecified |
| T78.3 | Angioneurotic oedema |
| T78.4 | Allergy, unspecified |
| T78.8 | Other adverse effects, not elsewhere classified |
| T78.9 | Adverse effect, unspecified |
| T80* | Complications following infusion, transfusion and therapeutic injection |
| T88.3 | Malignant hyperthermia due to anaesthesia |
| T88.6 | Anaphylactic shock due to adverse effect of correct drug or medicament properly administered |
| T88.7 | Unspecified adverse event of drug or medicament |
| Y40* | Drugs, medicaments, and biological substances causing adverse effects in therapeutic use - systemic antibiotic |
| Y41* | Drugs, medicaments, and biological substances causing adverse effects in therapeutic use - other systemic anti-infectives and antiparasitics |
| Y42* | Drugs, medicaments, and biological substances causing adverse effects in therapeutic use - Hormones and their synthetic substitutes and antagonists, not elsewhere classified |
| Y43* | Drugs, medicaments and biological substances causing adverse effects in therapeutic use - Primarily systemic agents |
| Y44* | Drugs, medicaments and biological substances causing adverse effects in therapeutic use - Agents primarily affecting blood constituents |
| Y45* | Drugs, medicaments and biological substances causing adverse effects in therapeutic use - Analgesics, antipyretics and anti-inflammatory drugs |
| Y46* | Drugs, medicaments and biological substances causing adverse effects in therapeutic use - Antiepileptics and antiparkinsonism drugs |
| Y47* | Drugs, medicaments and biological substances causing adverse effects in therapeutic use - Sedatives, hypnotics and antianxiety drugs |
| Y48* | Drugs, medicaments and biological substances causing adverse effects in therapeutic use - Anaesthetics and therapeutic gases |
| Y49* | Drugs, medicaments and biological substances causing adverse effects in therapeutic use - Psychotropic drugs, not elsewhere classified |
| Y50* | Drugs, medicaments and biological substances causing adverse effects in therapeutic use - Central nervous system stimulants, not elsewhere classified |
| Y51* | Drugs, medicaments and biological substances causing adverse effects in therapeutic use - Drugs primarily affecting the autonomic nervous system |
| Y52* | Drugs, medicaments and biological substances causing adverse effects in therapeutic use - Agents primarily affecting the cardiovascular system |
| Y53* | Drugs, medicaments and biological substances causing adverse effects in therapeutic use - Agents primarily affecting the gastrointestinal system |
| Y54* | Drugs, medicaments and biological substances causing adverse effects in therapeutic use - Agents primarily affecting water-balance and mineral and uric acid metabolism |
| Y55* | Drugs, medicaments and biological substances causing adverse effects in therapeutic use - Agents primarily acting on smooth and skeletal muscles and the respiratory system |
| Y56* | Drugs, medicaments and biological substances causing adverse effects in therapeutic use - Topical agents primarily affecting skin and mucous membrane and ophthalmological, otorhinolaryngological and dental drugs |
| Y57* | Drugs, medicaments and biological substances causing adverse effects in therapeutic use -Other and unspecified drugs and medicaments |
| Y59* | Drugs, medicaments and biological substances causing adverse effects in therapeutic use - Other and unspecified vaccines and biological substances |
| Y88.0 | Sequelae of adverse effects caused by drugs, medicaments and biological substances in therapeutic use |
| Z03.6 | Observation for suspected toxic effect from ingested substance |
| ***Poisoning by drug*** | |
| F55 | Abuse of non-dependence-producing substances |
| T36* | Poisoning by systemic antibiotics |
| T37* | Poisoning by other systemic anti-infectives and anti-parasitics |
| T38* | Poisoning by hormones and their synthetic substitutes and antagonists, not elsewhere classified |
| T39* | Poisoning by nonopioid analgesics, antipyretics and antirheumatics |
| T40* | Poisoning by narcotics and psychodysleptics |
| T41* | Poisoning by anaesthetics and therpeutic gases |
| T42* | Poisoning by antiepileptic, sedative-hypnotic and antiparkinsonism drugs |
| T43* | Poisoning by psychotropic drugs, not elsewhere classified |
| T44* | Poisoning by drugs primarily affecting the autonomic nervous system |
| T45* | Poisoning by primarily ststemic and haematological agents, not elsewhere classified |
| T46* | Poisoning by agents primarily affecting the cardiovascular system |
| T47* | Poisoning by agents primarily affecting the gastrointestinal system |
| T48* | Poisoning by agents primarily acting on smooth and skeletal muscles and the respiratory system |
| T49* | Poisoning by topical agents primarily affecting skin and mucous membrane and by ophthalmological, otorhinolaryngological and dental drugs |
| T50* | Poisoning by diuretics and other unspecified drugs, medicaments and biological substances |
| T96* | Sequelae of poisoning by drugs, medicaments and biological substances |
| X40* | Accidental poisoning by and exposure to nonopioid analgesics, antipyretics, and antirheumatics including acetominophen |
| X41* | Accidental poisoning by and exposure to antiepileptic, sedative hypnotic, antiparkinsonism, psychotropic drugs NEC |
| X42* | Accidental poisoning by and exposure to narcotics and psychodysleptics (hallucinogens) NEC (includes diamorphine (heroin), cocaine, codeine, lysergide (LSD), morphine, pethidine, narcotic NEC, hallucinogen NEC, hydrocodone, oxycodone (oxycontin) and fentanyl, among others) |
| X43* | Accidental poisoning by and exposure to other drugs acting on the autonomic nervous system |
| X44* | Accidental poisoning by and exposure to other and unspecified drugs, medicaments, and biological substances |
| Y10* | Poisoning by and exposure to nonopioid analgesics, antipyretics and antirheumatics, undetermined intent |
| ***Vaccine-associated*** | |
| A80.0 | Acute paralytic poliomyelitis, vaccine-associated |
| M02.2 | Postimmunization arthropathy |
| T88.0 | Infection following immunization |
| T88.1 | Infection complications following immunization |
| Y58* | Drugs, medicaments and biological substances causing adverse effects in therapeutic use - Bacterial vaccines |
| ***ADE very likely*** | |
| A04.7 | Enterocolitis due to Clostridium difficile |
| E15 | Nondiabetic hypoglycaemic coma |
| H91.0 | Ototoxic hearing loss |
| K52.1 | Toxic gastroenteritis and colitis |
| K71* | Toxic liver disease |
| L51* | Erythema multiforme |
| L56.2 | Photocontact dermatitis [berloque dermatitis] |
| N99.0 | Postprocedural renal failure |
| O74.2 | Cardiac complications of anaesthesia during labour and delivery |
| O74.3 | Central nervous system complications of anaesthesia during labour and delivery |
| Y63* | Failure in dosage during surgical and medical care |
| Y65.1 | Wrong fluid in infusion |
| Y69 | Unspecified misadventure during surgical and medical care |

ADE, adverse drug event; NEC, not elsewhere classified

(*) denote including sub-codes.
